# The effects of improved sanitation on diarrheal prevalence, incidence, and duration in children under five in the SNNPR State, Ethiopia: study protocol for a randomized controlled trial

**DOI:** 10.1186/s13063-016-1319-z

**Published:** 2016-04-18

**Authors:** Sunghoon Jung, Young-Ah Doh, Dawit Belew Bizuneh, Habtamu Beyene, Jieun Seong, Hyunjin Kwon, Yongwhan Kim, Girma Negussie Habteyes, Yigrem Tefera, Seungman Cha

**Affiliations:** Re-shaping Development Institute, 5 Yangpyeong-ro 12ga-gil, Yeongdeungpo-gu, Seoul, Republic of Korea; Korea International Cooperation Agency, 825 Daewangpangyo-ro, Sujeong-gu, Seongnam-si, Gyeongo-do 13449 Republic of Korea; BDS Center for Development Research, P.O. Box: 170609, CMC Road, Addis Ababa, Ethiopia; Health Bureau, Southern Nations, Nationalities, and Peoples’ Region, P.O. Box 149, Hawasa, Ethiopia; Department of Disease Control, Faculty of Infectious and Tropical Disease, London School of Hygiene & Tropical Medicine, Keppel Street, WC1E 7HT London, UK

**Keywords:** Community-Led Total Sanitation, Improved latrine, Effect, Diarrhea, Children under five

## Abstract

**Background:**

Diarrhea is one of the leading causes of death, killing 1.3 million in 2013 across the globe, of whom, 0.59 million were children under 5 years of age. Globally, about 1 billion people practice open defecation, and an estimated 2.4 billion people were living without improved sanitation facilities in 2015. Much of the previous research investigating the effect of improved sanitation has been based on observational studies. Recent studies have executed a cluster-randomized controlled trial to investigate the effect of improved sanitation. However, none of these recent studies achieved a sufficient level of latrine coverage. Without universal or at least a sufficient level of latrine coverage, a determination of the effect of improved latrines on the prevention of diarrheal disease is difficult.

This cluster-randomized trial aims to explore the net effect of improved latrines on diarrheal prevalence and incidence in children under five and to investigate the effect on the diarrheal duration.

**Method/design:**

A phase-in and factorial design will be used for the study. The intervention for improving latrines will be implemented in an intervention arm during the first phase, and the comparable intervention will be performed in the control arm during the second phase. During the second phase, a water pipe will be connected to the *gotts* (villages) in the intervention arm. After the second phase is completed, the control group will undergo the intervention of receiving a water pipe connection. For diarrheal prevalence, five rounds of surveying will be conducted at the household level. The first four rounds will be carried out in the first phase to explore the effect of improved latrines, and the last one, in the second phase to examine the combined effects of improved water and sanitation. For documentation of diarrheal incidence and duration, the mother or caregiver will record the diarrheal episodes of her youngest child on the “Sanitation Calendar” every day. Of 212 *gotts* in the project area, 48 *gotts* were selected for the trial, and 1200 households with a child under 5 will be registered for the intervention or control arm. Informed consent from 1200 households will be obtained from the mother or caregiver in written form.

**Discussion:**

To our knowledge, this is the second study to assess the effects of improved latrines on child diarrheal reduction through the application of Community-Led Total Sanitation.

**Trial registration:**

Current Controlled Trials, ISRCTN82492848

## Background

Diarrhea is one of the leading causes of death, killing 1.3 million in 2013, of whom 0.59 million were children under 5 years of age [1]. This preventable and curable disease accounts for 11 % of child mortality [2]. The lack of improved sanitation is the most important contributing factor to diarrheal disease in many low-income countries [3].

During the Millennium Campaign period, sanitation coverage has not progressed as planned and remains a daunting challenge for the next campaign of the Sustainable Development Goals [4, 5].

Globally, approximately 1 billion people practice open defecation, and an estimated 2.4 billion people lived without improved sanitation facilities in 2015 [1]. Sub-Saharan Africa showed slower progress in sanitation coverage, reaching 31 % in 2015 from 24 % in 1990, whereas South Asia has increased coverage to 49 % from 22 % in the same period [1].

Inequality in coverage also exists between rural and urban areas. In contrast with 40 % of the urban population accessing improved sanitation in sub-Saharan countries, only 23 % of people in rural areas have access to improved sanitation [1].

Ethiopia shows a high child mortality rate, with 74.4 children out of 1,000 live births dying before they reached the age of five in 2013. Diarrheal disease accounts for 9 % of child mortality, ranking as the fourth most frequent cause of child deaths [6]. In Ethiopia, 72 % of the people are living without improved sanitation facilities [7].

Much of the previous research investigating the effect of improved sanitation has been based on observational studies [8–11]. According to the results of a recent systematic review [12], few studies have involved a cluster-randomized controlled trial on sanitation intervention. Recently, several studies [13–15] have investigated the effect of improved sanitation, executing a cluster-randomized controlled trial. These studies did not demonstrate any protective effect of improved latrines against child diarrheal prevalence. However, none of the recent studies achieved universal or even a sufficient level of coverage required for fostering herd immunity. Without universal or at least a sufficient level of latrine coverage, the effect of improved latrines on the prevention of diarrheal disease is difficult to determine. Furthermore, most of the recent studies employing rigorous methodology have been conducted in South Asia. Since a number of people are living without improved sanitation in sub-Saharan Africa, a strong need exists for further evidence of the effect of improved sanitation in rural sub-Saharan Africa.

This study aims to explore the net effect of improved latrines on reducing diarrhea in children under five, particularly when latrine coverage reaches a certain threshold level. In addition, we aim to investigate the effect of improved latrines on the diarrheal duration of children by recording diarrheal incidence on a daily basis.

This study was designed as a cluster-randomized trial in the Southern Nations, Nationalities, and Peoples’ Region (the SNNPR State), Ethiopia, with the aim of finding evidence of the effect of improved latrines on diarrheal diseases in children under five. To our knowledge, this is the second study to assess the effects of improved latrines on child diarrheal reduction through the application of Community-Led Total Sanitation.

## Methods/design

### Study setting

**Fig. 1:**
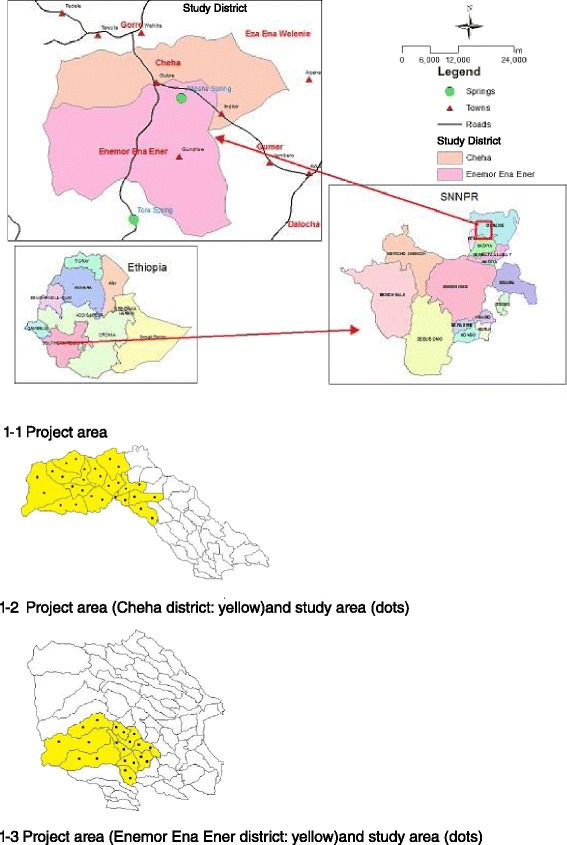
Project area and study area

The Cheha District and Enemore Ena Ener District, the target area of the project, are located 185 km southwest of Addis Ababa. According to the District Statistics Office, the total population of each district was 133,233 and 204,937, respectively, in 2014. Both districts are predominantly rural areas with 90 % of the entire land used for farming, and the major sources of income are crop production and livestock farming. Coffee, *chat*, and oil seeds are among the major cash crops in both districts, and plantations of eucalyptus trees for income are also very common. The dominant ethnic group, which accounts for more than 80 % of the population in the area, is the Gurage people, after which the administrative zone of the area is named (Gurage Zone). Of the population in the area, 64 % are Muslim, whereas 33 % identified themselves as Ethiopian Orthodox Christian (Fig. 1).

### Study design

The study was approved by the National Research Ethics Review Committee under the Ministry of Science and Technology, Federal Democratic Republic of Ethiopia (NRERC 3.10/032/2015; July 29, 2015) and was registered as an ISRCT on March 13, 2015 (ISRCTN82492848).

In the study, a *gott*, the Amharic word for village, was taken as the randomization unit because we expected that improved latrines could impact diarrheal transmission across households within a *gott*, where people interact with one another most closely. All the interventions related to latrine improvement will be performed at each *gott* level.

A phase-in and factorial design will be used for the study. The intervention for improving latrines will be implemented in the intervention arm during the first phase, and the comparable intervention will be performed in the control arm during the second phase. During the second phase, a water pipe will be connected to the *gotts* in the intervention arm. After the second phase is completed, the control group will receive the intervention of a water pipe connection. With the design, we will investigate not only the effect of improved sanitation but also the combined effects of improved water and sanitation interventions.

A preliminary survey was carried out to develop this water, sanitation, and hygiene (WaSH) project intervention design in March to May 2013. Water pipes will be connected to 212 *gotts*, of which, 48 *gotts* were selected as the study area of the trial. In the intervention *gotts*, the procedures of the Community-Led Total Sanitation including pre-triggering, triggering, and follow-up will be carried out for latrine improvement beginning in November 2015 and will continue to October 2016.

### Primary endpoint

The primary endpoint of the study is diarrheal prevalence in children under 5 years of age. We will use 7-day prevalence of reported diarrhea in the household, which will be based on parental reports. The survey will be conducted five times at the household level: the first four rounds will be carried out in the first phase to explore the effect of improved latrines, and the last one, in the second phase to assess the combined effects of improved water and sanitation.

**Fig. 2:**
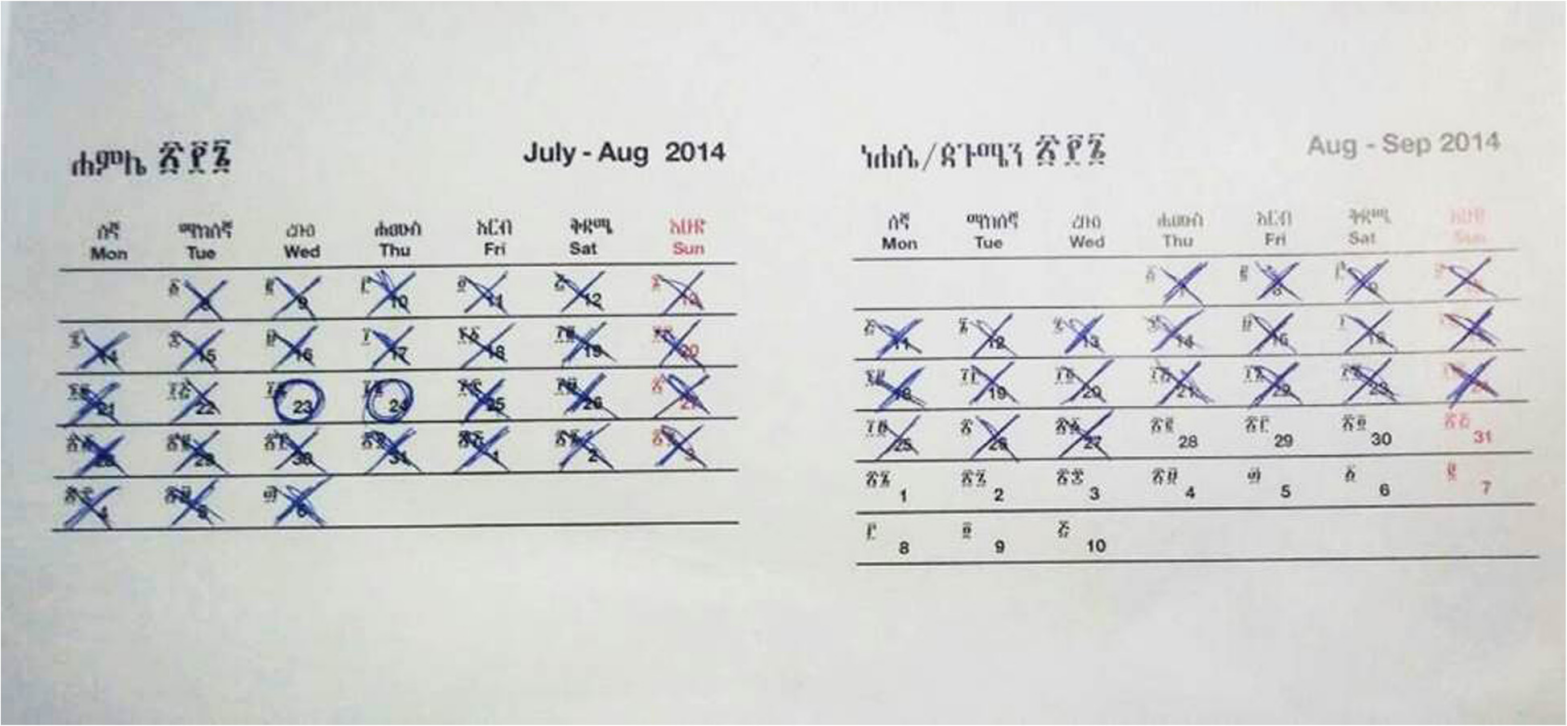
Sanitation calendar

In addition to diarrheal prevalence, we will measure diarrheal incidence and diarrheal duration. For documentation of diarrheal incidence and duration, we will have the mother or caregiver record the diarrheal episodes of her youngest child on a “Sanitation Calendar” (Amharic language: *Yenezehenna Gize Saleda*) on a daily basis (Fig. 2).

### Sample size calculation

#### Period prevalence of diarrhea

The prevalence of diarrhea was estimated to be 24 % on the basis of a preliminary survey in the SNNPR State, and we expect that our intervention will lead to a 30 % relative reduction on the basis of systematic review results [16]. Assuming a design effect of 2.14 and a coefficient of variation of 0.15, an 80 % study power resulted in 48 clusters (48 *gotts*) and 600 children per arm. We employed a two-stage cluster-sampling method for the study. Among the 212 *gotts* targeted by the project for the water pipe connection, 48 *gotts* were chosen as primary sampling units.

#### Incidence density of diarrhea in terms of child-weeks

The expected value of the incidence density, E (*s*^2^), is given by

$$ \mathrm{E}\left({\mathrm{s}}^2\right) = \lambda \mathrm{A}\mathrm{v}\left(1/{\mathrm{y}}_{\mathrm{j}}\right) + {\sigma^2}_{\mathrm{c}}=\lambda \mathrm{A}\mathrm{v}\left(1/{\mathrm{y}}_{\mathrm{j}}\right) + {\mathrm{k}}^2{\lambda}^2, $$

where λ is the true mean rate, *y*_*j*_ corresponds to the child-weeks of follow-up in the *j*^th^ cluster, Av() indicates the mean overall clusters, *σ*_*c*_^2^ is the between-cluster variance of the true rates, and *k* is the coefficient of variation of those rates [17]. Based on the preliminary survey, the overall diarrheal rate in the 48 *gotts* was 0.18 (or 18 cases per 100 child-weeks). The empirical standard deviation of the observed diarrhea rates was 0.092189, and the average of the reciprocal child-weeks per neighborhood was 0.001667. Therefore, *k* was estimated as

$$ \begin{array}{c}\hfill {\sigma}^2={0.092189}^2-0.18\times 0.001667=0.008199,\hfill \\ {}\hfill \hfill \\ {}\hfill \mathrm{and}\;\mathrm{therefore},\;k=\surd \left(0.008199/0.18\right)=0.213422.\hfill \end{array} $$

We assumed that the diarrhea rate in the control *gotts* remained constant at λ_0_ = 0.18, and we required 90 % power (*z*_*β*_ = 1.28) if the intervention reduced the diarrhea rate by 21 %. Assuming 600 child-weeks of observation (24 weeks of follow-up for 25 children) in each *gott*, the number of neighborhoods required for each treatment group is given by c = 1 + (1.96 + 0.28)^2^[(0.18 + 0.1422)/600 + 0. 213422^2^(0. 18^2^ + 0. 1422^2^)]/(0.18 − 0.1422)^2^ = 22.55.

### Household sampling methods

**Fig. 3:**
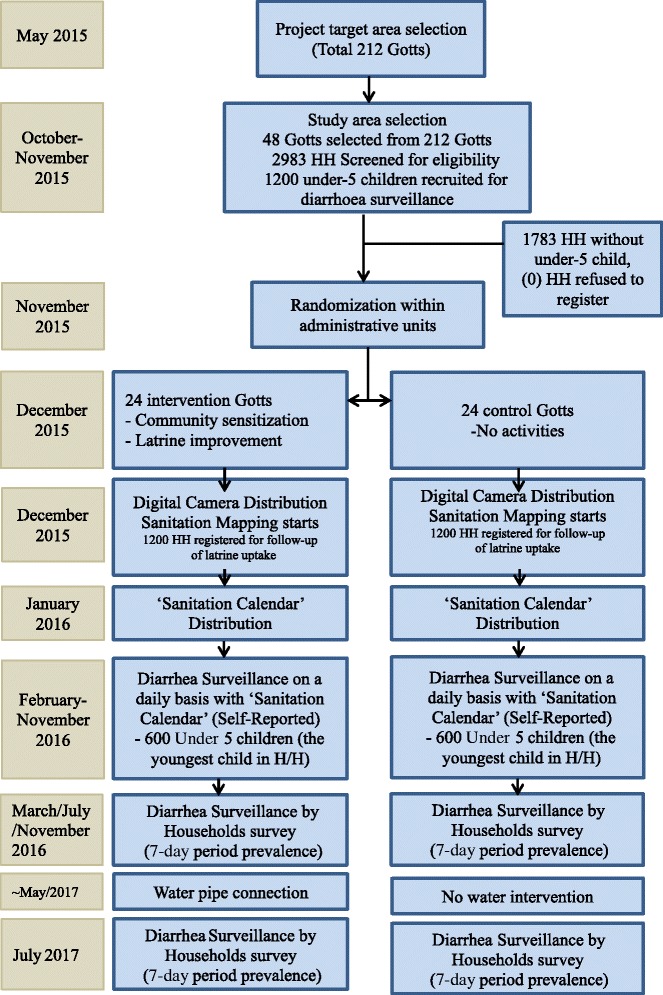
Flow diagram

A list of households with children under 5 years of age was established by four supervisors in each *gott* before the baseline survey. Supervisors required 8 days to produce a complete household list for all 48 *gotts*. Using SPSS 21 Statistics software, the survey team leader randomly sampled 25 households from each *gott*. Enumerators will recruit the youngest child under five from each household, from the fifth week of October through the second week of November 2015. If the mother or a household caregiver is absent at the time of the recruitment visit, the enumerator will revisit the same household two more times. If the mother or caregiver refused to be registered, a neighboring household with a child under 5 was visited to replace the household (Fig. 3).

### Eligibility criteria and randomization

The criteria for *gott*-level eligibility were (1) the lowest coverage of improved sanitation, (2) the lowest coverage of improved water, (3) similar population size, (4) similar distance from the main road, (5) road accessibility, (6) sufficient number of households with an under-five child, (7) sufficient distance between study *gotts* to prevent contamination, and (8) no other WaSH projects are to be implemented during the study period.

To select eligible *gotts* for the study, we used the data from the preliminary survey and from the special survey for the water pipe connection. The preliminary survey was carried out in March through May 2013, and the special survey was conducted in August 2014 in 212 *gotts* across the two target districts.

The criteria for household level eligibility are (1) having a child under 60 months at the time of recruitment and (2) agreeing to register with informed consent.

The age of a child will be verified with an immunization card, which shows the birth date of the child. Informed consent from 1200 households registered will be obtained from the mother or caregiver in written form. A structured questionnaire will be administered regarding water, sanitation, and hygiene, including demographics and socioeconomic characteristics.

The *gotts* in the study area were stratified according to the administrative unit (*kebelle*, in Amharic, which is the administrative unit above the *gott*) so that every *kebelle* contained both intervention and control *gotts*. By doing so, we were able to increase the balance between the intervention and control arms because *gotts* in the same *kebelle* were expected to have more common characteristics in terms of economic status, tribe, religion, geographical conditions such as altitude, and WaSH-related behaviors. In addition, local government officials of the study area also requested that we allocate the same number of study *gotts* in each *kebelle* to prevent any conflicts of interest among the *kebelles*. To avoid contamination, we allocated a buffer zone between the intervention and control *gotts* in every *kebelle*. A closed cohort design will be employed for the study. Any new baby born during the longitudinal survey period will not enrolled for the study.

### Intervention

In alignment with the National Hygiene and Sanitation Strategic Action Plan (2011-2015) [18], the Ethiopian Government’s policy on sanitation, we applied the principles of Community-Led Total Sanitation (CLTS) for the study. The Gurage zonal office, the SNNPR State of Ethiopia, and the Re-shaping Development Institute (ReDI) are implementing the project. The project is funded by the Korea International Cooperation Agency (KOICA).

### CLTS implementation

In accordance with the Ethiopian government’s guidelines on this issue, the primary approach for implementation of latrine improvement and hygiene promotion adopted for this program is Community-Led Total Sanitation. Of 48 *gotts* selected in the project area, 24 *gotts* in the project area were selected (intervention arm) and will receive the CLTS intervention and undergo intensive follow-up throughout the first phase of the implementation period (approximately 12 months) for latrine improvement. As the first step of CLTS, a team of trained CLTS facilitators will conduct the triggering process in each of these 24 *gotts*. During this triggering process, facilitators will use participatory tools, such as transect walk, sanitation mapping, and calculating feces deposition to help community members realize the health effects of open defecation practices in their *gotts*. In the process, basic human emotions, including shame and disgust, will be aroused among the *gott* members regarding their own defecation practices. Successfully implemented, the triggering activities of the CLTS, which normally take half a day for each *gott*, can be a very effective tool to motivate community members to stop open defecation and to eventually construct and use latrines of their own accord.

In accordance with the core principle of CLTS, no material or financial subsidies will be provided for the construction of individual household latrines. Household members will take responsibility for the whole process of latrine construction including (1) pit-hole digging; (2) constructing a slab and pit-hole cover; (3) constructing the walls, door, and roof; and (4) installing hand-washing facilities. The community member’s labor cost for construction of a latrine is expected to be approximately US$5.95 (125 Ethiopian birr as of October 23, 2015) per household. Ten full days of labor are estimated to be required for the construction of an improved latrine per household if two adults were to work together for the procurement of materials (wood, thatch, stone, and so on) and construction of the latrines.

**Fig. 4:**
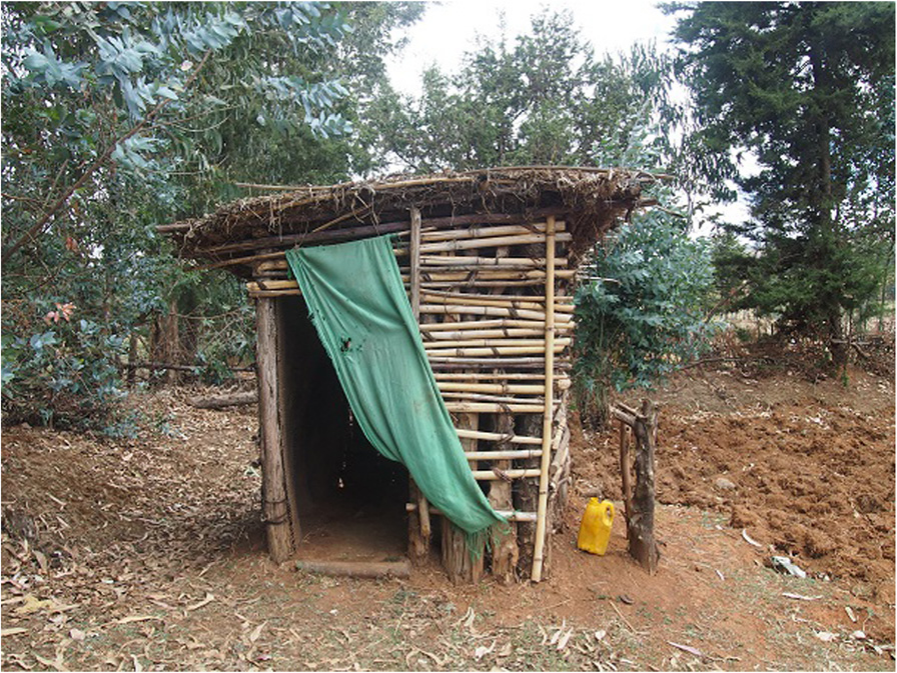
Model latrine

We produced an operational definition for an improved latrine for the project as having (1) a pit-hole with at least 2.5 m of depth, (2) a slab, (3) a pit-hole cover, (4) a superstructure, (5) a door, (6) a roof, and (7) hand-washing facilities (Fig. 4). We did not prescribe the materials for any component of the latrine, expecting those to be locally available and affordable materials to ensure sustainability. If a latrine lacks any of the elements specified above, it would not qualify as an improved latrine.

### WaSH promoter

At the beginning of the implementation period, one (1) villager from each intervention *gott* will be designated and trained as a WaSH promoter to carry out post-triggering or follow-up CLTS activities in his/her *gott*. To ensure the coverage and quality construction of the latrines in each *gott*, and also the long-term utilization of the latrines to be built, these WaSH promoters will employ a combination of various approaches including technical advice on latrine construction, collective awareness-raising, and household visits.

For this purpose, during the initial phase of implementation, WaSH promoters in each *gott* will be trained on key issues regarding latrine construction and use. Once trained, these WaSH promoters are to be the leading agents to promote and follow-up on the latrine improvement and sanitation-related behavior change in the promoter’s respective Gott. More specifically, WaSH promoters will be responsible for activities including household visits, community conversations, technical advice on latrine design, organization of and participation in monthly review meetings with stakeholders, and so on. In line with the Ethiopian government’s CLTS Guideline, no material subsidies will be provided for the construction of individual household latrines. Instead, most of the resources, both human and financial, of the project are to be allocated for activities aimed at promotion of latrine construction and use, as well as the sanitation behavior change of the community.

### Water intervention

When the CLTS intervention is completed, the second phase starts, during which water pipes will be connected to 24 *gotts* from one single spring on a mountain in each district. A spring capable of supplying sufficient water across the district is already identified and designing a water-pipe connection plan is under process. Immediately after the final round of the survey is finished, the water pipe will be connected to all the other 24 *gotts*.

### Sanitation mapping

To assess the progress of latrine uptake, a sanitation map will be drawn and updated on a monthly basis. In addition to the households recruited for the longitudinal household survey, all the households in the 48 *gotts* will be registered to assess the real-time latrine status. All the households in the 48 *gotts* will be endowed with an identity number and marked with red, yellow, or green. If a household completes construction of an improved latrine, the WaSH promoter will mark that household in green on the sanitation map. If the latrine is under construction, that household will be marked in yellow. If the uptake of an improved latrine does not occur, the household will be marked in red on the sanitation map. Each WaSH promoter will present the sanitation map for his/her *gott* during a monthly review meeting of WaSH promoters, which is intended to stimulate healthy competition between the *gotts*. A system of evaluation and reward at the *gott* level is established, and this system is expected to create peer-pressure within the *gott*. WaSH promoters will educate *gott* residents on the herd immunity effects [19] of improved latrines and the importance of universal or sufficient coverage of improved latrines. If a *gott* reaches 80 % improved latrine coverage, it will be certified as a “healthy *gott*” by the local government and will be rewarded by the project team. Material rewards will be given to both WaSH promoters individually and to those *gotts* collectively that show fast progress in improved latrine uptake.

### Scoring latrine improvement status by household

For the assessment of improved latrine coverage in each *gott*, only the households with improved latrines will be counted. Households not practicing open defecation, but using communal or neighbor’s latrines will not be counted in the latrine coverage assessment. To avoid subjectivity of WaSH promoters in categorizing the real-time status of latrine uptake, photos will be taken of a latrine by WaSH promoters and will be assessed and scored by supervisors and the team leader.

Direct observation will be made, especially of the latrine construction, utilization, and open defecation status. As for the latrine status, we will not only observe the presence of latrines but will also assess the latrine type for categorization by each element (e.g., pit more than 2.5 m deep, slab, pit-hole cover, superstructure/wall, roof, door, and hand-washing facility within 3 m of the latrine). To evaluate latrine utilization, odor, a spider web at the entrance, fresh feces, and a worn path to the latrine will be observed. To evaluate for ongoing open defecation, the presence of human feces around the household compound and in the *gott* will be observed.

In addition, to avoid measurement errors, 3-m-long sticks marked every 50 cm will be provided to WaSH promoters to measure the pit-hole depth. The presence of wet feces around the pit-hole and a spider-web at the entrance, and a worn path to the latrine will be observed to assess latrine utilization.

### Health outcome assessment with a sanitation calendar

We distributed a sanitation calendar to all registered households beginning in December 2015 and asked mothers or caregivers to mark “O” or “X” depending on the presence of diarrheal disease of her youngest child every day over the 12-month period of longitudinal observation from December 2015 to November 2016. WaSH promoters have been monitoring the recording status of the sanitation calendar on a weekly basis and continue to educate and encourage mothers or caregivers to continue recording appropriately.

We devised an incentive mechanism to encourage the mothers or caregivers to record diarrheal episodes on the sanitation calendar properly by establishing a system where a well-maintained calendar can be used as a voucher. A well-recorded calendar, regardless of whether it contains Os or Xs will be exchanged with a gift-in-kind, probably sanitation-related materials such as soaps, nail clippers, toothpastes, or the like. WaSH promoters will take a photo of the sanitation calendar using a mobile phone and submit to a supervisor and the team leader. For the study, diarrhea is defined as three or more watery stools in 24 hours according to the WHO definition.

### Intermediate outcomes

#### Sanitary survey

In each of the five rounds of the household survey (October 2015; March, July and November 2016; and July 2017), the presence of feces both inside and outside the household compound, and within the *gott* will be observed. The practices of child feces disposal will also be assessed by administering a structured questionnaire and observation.

#### Fly counts

**Fig. 5:**
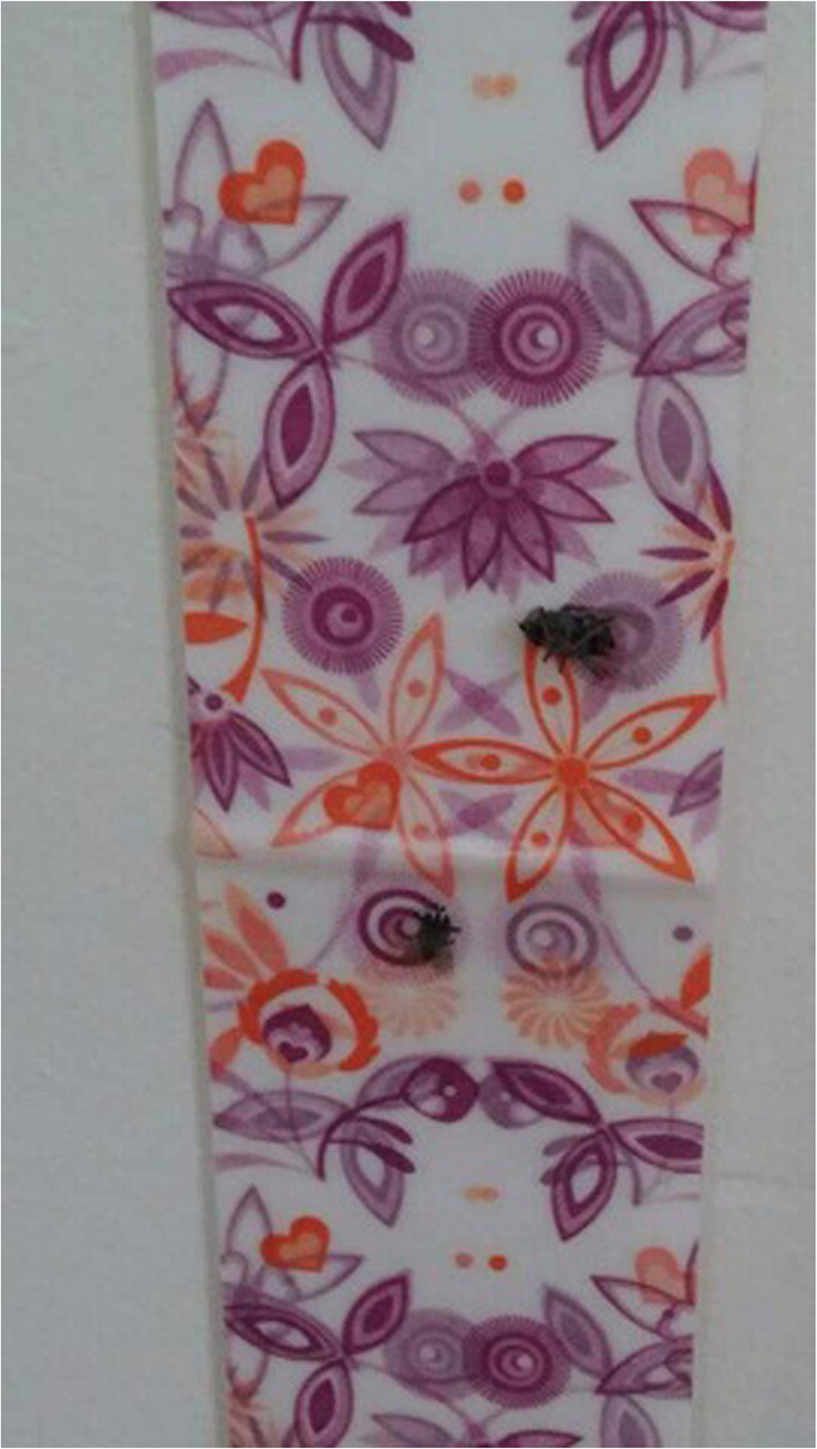
Fly trap

Flies are known to be key vectors transmitting diarrheal pathogens from human feces. We will count the number of flies with glue traps. For all five rounds of the household survey, enumerators will be provided with glue traps of the same length per each household, and they will hang them around the pit-hole of a latrine, if any, before starting the interview. The enumerators will count the number of flies stuck on the glue traps after 30 minutes (Fig. 5).

### Data analysis

We will conduct intention-to-treat analysis to assess the effects of improved latrines on child diarrheal reduction. For the main independent variables, the incidence density, 7-day recall period prevalence, and diarrheal duration will be calculated. We will use generalized estimating equations to investigate the effect at the cluster level and a log-binomial model to calculate the incidence rate of diarrhea. Taking into consideration the between-cluster variation by assuming that there are cluster-level effects, we will use the random effects model. We will also conduct a multilevel analysis to explore the effects of coverage of a *gott* on the diarrheal incidence of an individual. (Coverage is defined as low if it is below 33 %; medium, 33–66 %; and high, above 66 %).

## Discussion

This study seeks to assess the effects of improved sanitation on the health gains of children under 5 years of age and to explore the behavioral factors associated with latrine improvement. Several studies [13–15] have been conducted for a similar purpose with rigorous methodology in South Asian countries; however, those studies failed to reach universal or sufficient coverage of improved latrines. Furthermore, the results of recent randomized controlled trials cannot represent the effect of Community-Led Total Sanitation because they provided material or financial subsidies, although these were limited to the marginalized groups in the community. For example, a recent study [20] on improved sanitation, conducted in Bangladesh, revealed that subsidies could increase latrine ownership both in subsidized and unsubsidized households; however, those authors did not investigate the effects of the approaches for latrine improvement on scalability and sustainability, nor did they explore the effects on maintenance. For this study, we strictly applied the main principle of Community-Led Total Sanitation, paying close attention to the potential for scalability and sustainability. In the trial, community members will fully contribute to constructing their own household latrines, including digging a pit-hole; obtaining materials for the slab, pit-hole cover, walls, and roof; and completing installation.

For the study, a sanitation calendar has been devised to track child diarrhea more efficiently. We estimate the incidence density of diarrhea. With the sanitation calendar, the potential of recall bias [21] and respondent fatigue [22] due to frequent visits, which were the limitations of previous studies, are expected be reduced. Moreover, the sanitation calendar will help us to investigate the effects of improved sanitation on the duration of diarrheal diseases through the daily recording of diarrheal episodes. Assessing the effects of improved sanitation on the diarrheal duration has continuously been recommended as a future study topic in previous studies.

To ensure objectivity and avoid information bias, latrine improvement status will be photographed with tablet PCs or mobile phones provided to enumerators and WaSH promoters. Assessments of the latrine improvement status will be made by supervisors and team leaders, not by the enumerators or WaSH promoters, which helps avoid overestimating the coverage of improved latrines. Latrine improvement status will be scored on the basis of the photos taken. Using the scored results, the degree of the effects of the improved latrines will be explored according to the improved status.

To avoid contamination between the intervention and control *gotts* in the same *kebelle*, we excluded any neighboring *gotts* of either intervention or control *gotts* from selection, thus making those *gotts* serve as a buffer zone [23]. In this trial, keeping records on the sanitation calendar is one of the most important features. In order to encourage mothers or caregivers to record diarrheal incidence every day, WaSH promoters will visit registered households on a weekly basis. The sanitation calendar will be photographed on this visit, and the supervisors and team leader will randomly revisit some of the households to verify the results. The system of encouragement and verification will be strictly maintained, particularly during the first 2 months of the intervention period, so the mothers or caregivers of registered households will be familiar with recording cases of diarrhea. A community meeting will be convened every month, and the WaSH promoter will correct substantial errors related to sanitation calendar utilization.

Our intervention is not to provide any financial nor material subsidy to community members. Rather, our intervention is to encourage community members to construct latrines for their own household members using locally available materials. We strictly comply with the key principles of Community-Led Total Sanitation (CLTS). Therefore, like all the previous studies, we have similar limitations such as the possibility of insufficient coverage.

This study will provide sound evidence for determining the effects of improved latrines on the prevalence, incidence density, and duration of diarrheal disease of children under five.

### Trial status

Trial recruitment had not commenced as of October 23, 2015.

## Abbreviations

CLTSH: Community-Led Total Sanitation and Hygiene
MDGs: Millennium Development Goals
NRERC: National Research Ethics Review Committee
SNNPR: Southern Nations, Nationalities, and Peoples’ Region
WaSH: Water, Sanitation, and Hygiene.
